# Trends Over Time in Use of Nonrecommended Tests and Treatments Since Publication of the American Academy of Pediatrics Bronchiolitis Guideline

**DOI:** 10.1001/jamanetworkopen.2020.37356

**Published:** 2021-02-15

**Authors:** Samantha A. House, Jennifer R. Marin, Matthew Hall, Shawn L. Ralston

**Affiliations:** 1 Department of Pediatrics, Children’s Hospital at Dartmouth-Hitchcock Medical Center, Lebanon, New Hampshire; 2 Department of Pediatrics, Geisel School of Medicine, Dartmouth College, Hanover, New Hampshire; 3 UPMC Children’s Hospital of Pittsburgh, Pittsburgh, Pennsylvania; 4 Children’s Hospital Association, Lenexa, Kansas; 5 Department of Pediatrics, Johns Hopkins Medical School, Baltimore, Maryland

## Abstract

**Importance:**

Acute viral bronchiolitis is a common and costly pediatric condition for which clinical practice guidelines discourage use of diagnostic tests and therapies.

**Objective:**

To evaluate trends over time for use of nonrecommended services for bronchiolitis since publication of the American Academy of Pediatrics clinical practice guideline on bronchiolitis (originally published in October 2006 and updated in November 2014).

**Design, Setting, and Participants:**

This cohort study was conducted using interrupted time-series regression analysis adjusting for the hospital providing service, patient demographic characteristics, and payer, with 2014 guideline update publication as the event point. Included patients were children younger than 2 years old discharged from the emergency department (ED) or hospital inpatient setting with a primary diagnosis of bronchiolitis at US Children’s Hospitals contributing data to the Pediatric Health Information Systems database. Data were analyzed from June through December 2020.

**Main Outcomes and Measures:**

Rates of nonrecommended tests (ie, chest radiography, viral testing, and complete blood cell count) and treatments (ie, bronchodilators, corticosteroids, antibiotics) were measured.

**Results:**

Among 602 375 encounters involving children with a primary diagnosis of bronchiolitis, 404 203 encounters (67.1%) were ED discharges and 198 172 encounters (32.9%) were inpatient discharges; 468 226 encounters (77.7%) involved children younger than 12 months, and 356 796 encounters (59.2%) involved boys. In the period after initial guideline publication (ie, November 2006 to November 2014), a negative use trajectory was found in all measures except viral testing in the ED group. Using the 2014 guideline update as the event point, several measures showed decreased use between study time periods. The greatest decrease was in bronchodilator use, which changed by −13.5 percentage points in the ED group (95% CI, −15.2 percentage points to −11.8 percentage points) and −11.3 percentage points in the inpatient group (95% CI, −13.1 percentage points to −9.4 percentage points). In the period after the 2014 guideline update (ie, December 2014 to December 2019), bronchodilators also showed the greatest change in usage trajectory, steepening more than 2-fold in both groups. In the ED group, the negative trajectory steepened from −0.11% monthly (95% CI, −0.13% to −0.09%) in the first guideline period to a new mean monthly slope of −0.26% (95% CI, −0.30% to −0.23%). In the inpatient group, the mean monthly slope steepened from −0.08% (95% CI, −0.10 to −0.05%) to −0.26% (95% CI, 0.30% to −0.22%). Length of stay decreased from 2.0 days (95% CI, 1.9 days to 2.1 days) to 1.7 days (95% CI, 1.7 days to 1.8 days). Hospital admission rate decreased from 18.0% (95% CI, 13.8% to 22.2%) to 17.8% (95% CI, 13.6 to 22.1%).

**Conclusions and Relevance:**

This cohort study with interrupted time-series analysis found that use of most nonrecommended bronchiolitis services decreased continuously after 2006. The rate of decline in bronchodilator use increased more than 2-fold after the 2014 guideline update. These findings support potential associations of practice guidelines with improved bronchiolitis care.

## Key Points

**Question:**

How has use of nonrecommended tests and treatments for bronchiolitis changed since publication of the 2006 American Academy of Pediatrics (AAP) clinical practice guideline?

**Findings:**

This cohort study of 602 375 bronchiolitis encounters found decreased use of most nonrecommended services for bronchiolitis in a sample of US children’s hospitals over the 13 years since the original AAP guideline publication. Trend changes were greatest in bronchodilator use, with a significantly steepening decrease in trajectory occurring after publication of the 2014 guideline update.

**Meaning:**

These findings suggest that clinical practice guidelines may contribute to clinical improvements in bronchiolitis care.

## Teaser

This cohort study examines changes in use of nonrecommended tests and treatments among children with bronchiolitis after publication of the American Academy of Pediatrics Clinical Practice Guideline.

## Introduction

Bronchiolitis is a common pediatric illness with a robust evidence base suggesting that no specific test or treatment is associated with a change in the clinical disease course.^1^ Despite this, the delivery of low-value care to children with bronchiolitis persists.^2,3^ This is associated with increased risk for adverse outcomes from unnecessary care and increased health care costs.^1,4^ Consequently, bronchiolitis is the target of many international guidelines aiming to standardize care.^5^ In the United States, the American Academy of Pediatrics (AAP) published a clinical practice guideline in 2006^6^ and updated it in 2014.^1^ The most substantive change to the 2014 guideline^1^ was a recommendation against beta-agonist use among children with bronchiolitis, replacing support in the 2006 guideline^6^ for a monitored trial.

Several studies have assessed clinical care outcomes following publication of the 2006 AAP bronchiolitis guideline using different methods and databases. Comparing care delivered in the post-2006 guideline period (ie, 2006-2012) with the preguideline period, Parikh et al^7^ found reduced use of bronchodilators, corticosteroids, complete blood counts (CBCs), respiratory syncytial virus testing, and chest radiographs (CRs) among patients admitted to the hospital.^7^ Employing a similar before and after study design to assess emergency department (ED) care, Johnson et al^8^ found a reduction in CR use only, with no other changes in the period after guideline publication (ie, 2006-2009). Assessing changes in compliance with guideline recommendations in the postguideline period, Florin et al^2^ found decreasing rates of corticosteroid and CR use from 2007 to 2012 among patients admitted to the hospital, whereas Burstein et al^9^ found no change in rates of CR use and Papenburg et al^10^ found no change in rates of antibiotic use in the ED from 2007 to 2015.

Thus, current evidence on provider compliance with AAP bronchiolitis guideline recommendations may be incomplete. Questions remain surrounding the amount of change associated with guideline publication and the degree to which any improvements associated with guideline publication may be sustained over time. Furthermore, none of the existing literature, to our knowledge, addresses a significant time period following the 2014 update, leaving important knowledge gaps surrounding the utility of guideline updates. In this study, we aimed to evaluate trends over time in use of nonrecommended tests and treatments for bronchiolitis from the time of the 2006 AAP guideline publication to the present among a sample of patients in US children’s hospitals.

## Methods

The Dartmouth-Hitchcock Medical Center Institutional Review Board approved this cohort study upon determination that it did not constitute human subjects research. The study was conducted in accordance with Strengthening the Reporting of Observational Studies in Epidemiology (STROBE) reporting guideline.

### Study Design and Data Source

This was a retrospective, observational hospital cohort study using the Pediatric Health Information Systems (PHIS) database (Children’s Hospital Association). The PHIS database contains deidentified administrative data from a large sample of tertiary care children’s hospitals. The database accounts for approximately 20% of pediatric hospitalizations in the United States. Data quality is ensured through a joint effort between the Children’s Hospital Association and participating hospitals.

### Patient Population

We included encounters by children ages 28 days to 2 years discharged from November 1, 2006, to December 31, 2019, with a primary discharge diagnosis of acute bronchiolitis (*International Classification of Diseases, Ninth Revision*^11^ code 466.11 or 466.19 or *International Classification of Diseases, Tenth Revision*^12^ code J21.X). We created 2 patient groups for analysis in this study: the ED group and the inpatient group.

#### ED Group

In PHIS, an encounter is defined as *ED* if the patient was ultimately discharged from the ED and not admitted to the hospital. Therefore, our ED group included only children discharged from the ED; all measured outcomes were reflective of care administered in the ED setting.

#### Inpatient Group

For children admitted to a hospital, PHIS combines billing for care delivered during the associated ED encounter with billing data for that hospitalization. Therefore, our inpatient group included usage from the associated ED visit as well as inpatient charges (except in the case of a transfer from an outside ED). Inpatient encounters were included only if the encounter also received the All Patient Refined Diagnosis Related Groups (version 36) for bronchiolitis and respiratory syncytial virus pneumonia (code 138) for specificity. Observation encounters were included in the inpatient group.

Only hospitals contributing data for the full study period were included. This was determined by setting, such that hospitals must have contributed ED data for the full study period for their data to be included in the ED group and inpatient data for the full study period for their data to be included in the inpatient group. We excluded encounters by patients with complex chronic conditions,^13^ those with a billing charge for mechanical ventilation, and those with an inpatient length of stay (LOS) of greater than 10 days as these encounters represent clinical scenarios that fall outside those for which current clinical guidelines offer recommendations.^1^ All encounters meeting inclusion criteria were analyzed individually. We did not exclude hospital readmissions or repeat ED visits within the same episode of illness; these were treated as distinct clinical encounters.

Encounters were grouped into 2 periods based on discharge date. Guideline period 1 spanned from November 2006, 1 month after the publication of the 2006 AAP guideline, to November 2014, the month of the 2014 guideline publication. Guideline period 2 spanned from December 2014, 1 month after the publication of the 2014 guideline, to December 2019.

### Measures

The primary outcomes were rates of diagnostic testing and treatment as determined from billing data. We included tests (ie, CBC, CR, and viral testing) and treatments (ie, bronchodilators, corticosteroids, and antibiotics) that the AAP guidelines specifically recommend against in the evaluation and management of routine bronchiolitis.^1^ Bronchodilators included any form of epinephrine, albuterol, or levalbuterol. We analyzed LOS for inpatient and observation encounters and hospital admission rates from the ED between the 2 guideline periods as balancing measures.

### Statistical Analysis

Demographic characteristics were summarized with frequencies and percentages, with values from guideline period 1 compared with those from guideline period 2 using χ^2^ tests to identify candidate variables for adjustment. Segmented regression analysis with an interrupted time series (ITS) was conducted with the publication of the 2014 guideline as the event point. This analysis measured rates of change in outcomes over the 2 study periods, defined as the slope associated with use of tests and treatments over each period, and level change, defined as the change in use of tests and treatments between time periods divided by a single event point. Monthly rates of resource use were included in the segmented regression analysis. The model adjusted for the hospital providing service with random intercepts to control for clustering, as well as fixed effects for age, race/ethnicity, and payer. The ITS output is presented as a point estimate with 95% CIs for monthly slopes, change in slope between time periods, and level change between time periods.

All statistical analyses were performed using SAS version 9.4 (SAS Institute) from June through December 2020. Changes were considered statistically significant if the 95% CI did not include 0.

## Results

### Patient Population

Over the 2006 to 2019 study period, 25 hospitals contributed complete data for the ED group and 35 hospitals contributed complete data for the inpatient group. There were 602 375 encounters among children discharged with a primary diagnosis of bronchiolitis; 404 203 encounters (67.1%) were in the ED group and 198 172 encounters (32.9%) were in the inpatient group. In both groups and guideline periods, 468 226 encounters (77.7%) involved children 12 months of age or younger, 356 796 encounters (59.2%) involved boys, and 223 098 encounters (37.0%) involved non-Hispanic White patients. Race/ethnicity differences were greatest in the inpatient setting, where 80 234 encounters (40.5%) involved non-Hispanic White patients, compared with 142 864 encounters (35.3%) in the ED setting (Table 1).

**Table 1.** Patient Characteristics by Group

Characteristic, No. (%)	ED group (25 hospitals)		Inpatient group (35 hospitals)	
	Guideline period 1 (N = 211 402)	Guideline period 2 (N = 192 801)	Guideline period 1 (N = 105 524)	Guideline period 2 (N = 92 648)
Age				
28 d to <3 mo	30 810 (14.6)	25 491 (13.2)	34 291 (32.5)	22 329 (24.1)
≥3 mo to <6 mo	64 013 (30.3)	53 558 (27.8)	24 530 (23.2)	20 217 (21.8)
≥6 mo to <12 mo	73 050 (34.6)	67 684 (35.1)	26 820 (25.4)	25 433 (27.5)
≥12 mo to 2 y	43 529 (20.6)	46 068 (23.9)	19 883 (18.8)	24 669 (26.6)
Girls	86 049 (40.7)	78 664 (40.8)	43 398 (41.1)	37 468 (40.4)
Race/ethnicity				
Non-Hispanic				
White	72 608 (34.3)	70 256 (36.4)	40 645 (38.5)	39 589 (42.7)
Black	62 130 (29.4)	57 366 (29.8)	25 075 (23.8)	21 188 (22.9)
Hispanic	55 565 (26.3)	46 335 (24.0)	28 749 (27.2)	21 076 (22.7)
Asian	2902 (1.4)	4016 (2.1)	2182 (2.1)	2596 (2.8)
Other^a^	18 197 (8.6)	14 828 (7.7)	8873 (8.4)	8199 (8.8)
Payer				
Government	148 638 (70.3)	132 408 (68.7)	69 377 (65.7)	59 585 (64.3)
Private	51 112 (24.2)	53 772 (27.9)	30 142 (28.6)	30 453 (32.9)
Other^b^	11 652 (5.5)	6621 (3.4)	6005 (5.7)	2610 (2.8)

Abbreviation: ED, emergency department.

^a^ Includes Hawaiian/Pacific Islander, American Indian or Alaska Native, multirace, other, and unknown.

^b^ Includes self-pay, charity, hospital chose not to bill for encounter, unknown, and other.

### Trends in Testing

Over guideline period 1 (ie, November 2006 to November 2014), the ITS model found a decrease in use for all testing measures in both groups, with the exception of viral testing in the ED group (Table 2). In the ED group, the percent of encounters with CBC use changed by a mean of −0.03% monthly (95% CI, −0.04% to −0.02%) and the percent of encounters with CR use changed by a mean of −0.19% monthly (95% CI, −0.21% to −0.18%). In the inpatient group, the percent of encounters with CBC use changed by a mean of −0.13% monthly (95% CI, −0.16% to −0.11%), the percent with CR use changed by a mean of −0.23% monthly (95% CI, −0.26 to −0.21), and the percent with viral testing changed by a mean of −0.13% monthly (95% CI, −0.16% to −0.11%).

**Table 2.** Usage Trends in Guideline Nonrecommended Tests and Treatments

Service	Guideline period 1 (2006-2014)			Guideline period 2 (2014-2019)			Change between guideline periods	
	Adjusted use, % (95% CI)		Monthly slope (95% CI)	Adjusted use, % (95% CI)		Monthly slope (95% CI)	Change in slope (95% CI)^a^	Level change (95% CI)^b^
	Start	End		Start	End			
**Testing**								
ED group								
Complete blood count	6.4 (5.1-7.7)	3.7 (2.4-5.0)	−0.03 (−0.04 to −0.02)	4.0 (2.7-5.3)	2.3 (1.0-3.6)	−0.02 (−0.03 to −0.003)	0.01 (−0.01 to 0.027)	0.3 (−0.3 to 1.0)
Chest radiograph	48.4 (44.0-52.9)	30.2 (25.9-34.5)	−0.19 (−0.21 to −0.18)	25.9 (21.5-30.2)	14.2 (9.8-18.7)	−0.13 (−0.17 to −0.09)	0.06 (0.02 to 0.10)	−4.1 (−5.9 to −2.4)
Viral testing	23.5 (16.2-30.8)	22.6 (15.3-29.8)	−0.01 (−0.04 to 0.01)	18.9 (11.6-26.1)	18.3 (11.0-25.6)	−0.04 (−0.09 to −0.01)	−0.03 (−0.08 to 0.03)	−3.7 (−5.9 to −1.5)
Inpatient group								
Complete blood count	44.6 (39.8-49.3)	32.2 (27.6-36.8)	−0.13 (−0.16 to −0.11)	31.5 (26.8-36.1)	23.6 (18.8-28.3)	−0.07 (−0.12 to −0.03)	0.07 (0.02 to 0.11)	−0.8 (−2.7 to 1.1)
Chest radiograph	78.8 (75.6-82.1)	57.3 (54.2-60.4)	−0.23 (−0.26 to −0.21)	51.1 (47.9-54.2)	37.3 (34.0-40.6)	−0.23 (−0.27 to −0.19)	0.01 (−0.04 to 0.06)	−6.3 (−8.1 to −4.4)
Viral testing	60.9 (55.3-66.6)	48.7 (43.1-54.2)	−0.13 (−0.16 to −0.11)	41.4 (35.8-46.9)	33.5 (27.8-39.2)	−0.11 (−0.16 to −0.05)	0.03 (−0.03 to 0.09)	−7.3 (−9.6 to −5.0)
**Treatment**								
ED group								
Bronchodilators	56.3 (51.1-61.5)	46.1 (41.0-51.2)	−0.11 (−0.13 to −0.09)	32.5 (27.3-37.7)	26.0 (20.8-31.2)	−0.26 (−0.30 to −0.23)	−0.15 (−0.20 to −0.11)	−13.5 (−15.2 to −11.8)
Steroids	15.7 (13.0-18.3)	11.1 (8.5-13.7)	−0.05 (−0.06 to −0.04)	9.0 (6.4-11.6)	6.1 (3.4-8.7)	−0.04 (−0.06 to −0.01)	0.01 (−0.02 to 0.04)	−2.0 (−3.1 to −0.9)
Antibiotics	4.2 (3.3-5.0)	3.6 (2.7-4.4)	−0.01 (−0.01 to −0.001)	3.1 (2.2-3.9)	2.7 (1.8-3.6)	0.01 (−0.004 to 0.02)	0.01 (0.001 to 0.03)	−0.5 (−1.0 to −0.01)
Inpatient group								
Bronchodilators	73.0 (68.0-78.0)	65.8 (60.9-70.7)	−0.08 (−0.10 to −0.05)	54.7 (49.8-59.6)	50.1 (45.1-55.1)	−0.26 (−0.30 to −0.22)	−0.18 (−0.23 to −0.13)	−11.3 (−13.1 to −9.4)
Steroids	38.6 (35.3-42.0)	29.8 (26.7-32.9)	−0.09 (−0.12 to −0.07)	23.6 (20.5-26.7)	17.9 (14.6-21.2)	−0.08 (−0.13 to −0.03)	0.01 (−0.04 to 0.07)	−6.2 (−8.5 to −3.9)
Antibiotics	49.1 (46.9-51.2)	34.0 (32.2-35.9)	−0.16 (−0.19 to −0.14)	33.4 (31.5-35.2)	23.8 (21.6-25.9)	−0.11 (−0.15 to −0.07)	0.06 (0.01 to 0.11)	−0.7 (−2.7 to 1.3)
Balancing								
Hospital admission rate	18.0 (13.8-22.2)	18.0 (13.8-22.2)	0.00 (−0.004 to 0.004)	17.9 (13.6-22.1)	17.8 (13.6-22.1)	0.02 (0.01 to 0.03)	0.02 (0.01 to 0.03)	−0.02 (−0.01 to 0.03)
Length of stay, d	2.0 (1.9-2.1)	1.8 (1.7-1.9)	−0.002 (−0.003 to −0.001)	1.9 (1.8-1.9)	1.7 (1.7-1.8)	−0.002 (−0.003 to 0.000)	0.001 (0 to 0.002)	0.03 (0.02 to 0.08)

Abbreviation: ED, emergency department.

^a^ Change in slope is defined as the difference in slope between guideline period 1 and guideline period 2.

^b^ Level change is defined as the change in usage rate between the 2 time periods (ie, start of guideline period 2 vs end of guideline period 1) using 2014 guideline publication as the event point.

Using the 2014 guideline publication as the event point for the ITS analysis, there were decreases in use of CR and viral testing between guideline periods. In the ED group, the percent of encounters with CR use changed by −4.1 percentage points (95% CI, −5.9 percentage points to −2.4 percentage points) between the 2 periods, and the percent with viral testing changed by −3.7 percentage points (95% CI, −5.9 percentage points to −1.5 percentage points). In the inpatient group, the percent with CR use changed by −6.3 percentage points (95% CI, −8.1 percentage points to −4.4 percentage points) and the percent with viral testing changed by −7.3 percentage points (95% CI, −9.6 percentage points to −5.0 percentage points) between periods (Table 2). Use of all testing measures continued to decrease over guideline period 2, although the decrease slowed for CR in the ED group and CBC in the inpatient group (Table 2).

**Figure 1.**
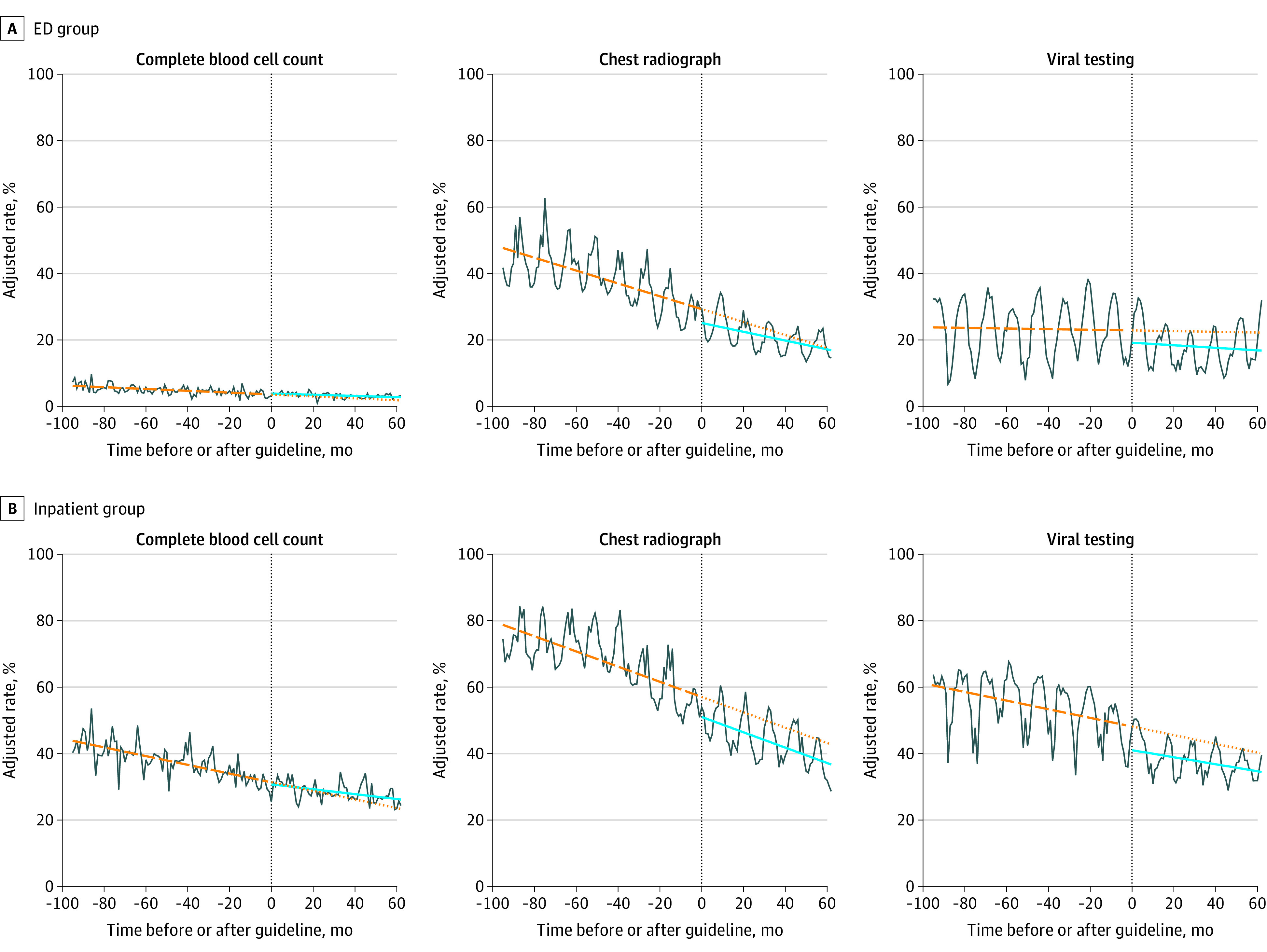
Trends in Testing Measures The vertical line in each panel indicates the division between guideline period 1 and guideline period 2; distance between the preguideline trajectory and postguideline trajectory at this transition point, the reported level change; dashed orange line, slope over guideline period 1; dotted orange line, projected slope over guideline period if no change had occurred at the event point; solid blue line, projected slope over guideline period 2; ED, emergency department.

Over the full study period, in the ED group, adjusted use decreased from 6.4% of encounters (95% CI, 5.1%-7.7%) to 2.3% (95% CI, 1.0%-3.6%) for CBC, 48.4% (95% CI, 44.0%-52.9%) to 14.2% (95% CI, 9.8%-18.7%) for CR, and 23.5% (95% CI, 16.2%-30.8%) to 18.3% (95% CI, 11.0% to 25.6%) for viral testing. In the inpatient group, adjusted use decreased from 44.6% of encounters (95% CI, 39.8%-49.3%) to 23.6% (95% CI, 18.8%-28.3%) for CBC, 78.8% (95% CI, 75.6%-82.1%) to 37.3% (95% CI, 34.0%-40.6%) for CR, and 60.9% (95% CI, 55.3%-66.6%) to 33.5% (95% CI, 27.8%-39.2%) for viral testing. Trends in testing measures over time are shown in Figure 1.

### Trends in Treatments

Over guideline period 1, the ITS model found decreasing use for all treatments in both group (Table 2). In the ED group, the percent of encounters with bronchodilator use changed by a mean of −0.11% monthly (95% CI, −0.13% to −0.09%), the percent with steroid use changed by a mean of −0.05% monthly (95% CI, −0.06 to −0.04%), and the percent with antibiotic use changed by a mean of −0.01% monthly (95% CI, −0.01% to −0.001%). In the inpatient group, the percent with bronchodilator use changed by a mean of −0.08% monthly (95% CI, −0.10% to −0.05%), the percent with steroid use changed by a mean of −0.09% monthly (95% CI, −0.12% to −0.07%), and the percent with antibiotic use changed by a mean of −0.16% monthly (95% CI, −0.19% to −0.14%).

Using the 2014 guideline publication as the event point for the ITS analysis, there were decreases in all treatment measures between guideline periods. In the ED group, the percent of encounters with bronchodilator use changed by −13.5 percentage points (95% CI, −15.2 percentage points to −11.8 percentage points), the percent with steroid use changed by −2.0 percentage points (95% CI, −3.1 percentage points to −0.9 percentage points), and the percent with antibiotic use changed by −0.5 percentage points (95% CI, −1.0 percentage points to −0.01 percentage points) between periods. In the inpatient group, the percent of encounters with bronchodilator use changed by −11.3 percentage points (95% CI, −13.1 percentage points to −9.4 percentage points) and the percent with steroid use changed by −6.2 percentage points (95% CI, −8.5 percentage points to −3.9 percentage points) between periods (Table 2).

Over guideline period 2, the decrease in bronchodilator use found in guideline period 1 steepened significantly (Table 2). In the ED group, the negative slope steepened by −0.15% (95% CI, −0.20 to −0.11), to a new mean monthly slope of −0.26% (95% CI, −0.30 to −0.23). In the inpatient group, the negative slope steepened by −0.18% (95% CI, −0.23 to −0.13), to a new mean monthly slope of −0.26% (95% CI, −0.30 to −0.22). Use of all treatment measures continued to decrease significantly across guideline period 2, except for antibiotics in the ED group; there was also a slowing of the declining trajectory in antibiotic use in the inpatient group over this period.

**Figure 2.**
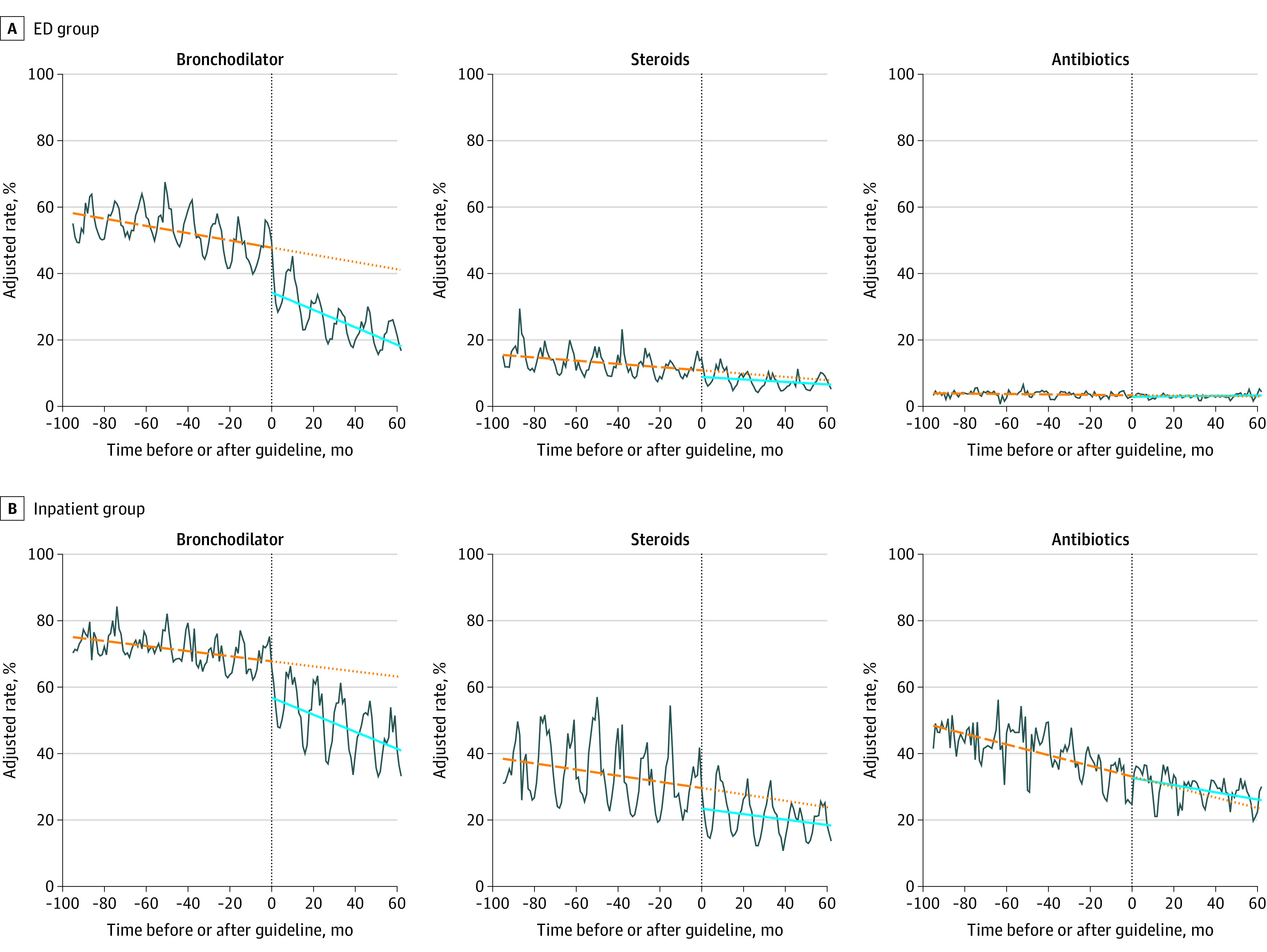
Trends in Treatment Measures The vertical line in each panel indicates the division between guideline period 1 and guideline period 2; distance between the preguideline trajectory and postguideline trajectory at this transition point, the reported level change; dashed orange line, slope over guideline period 1; dotted orange line, projected slope over guideline period if no change had occurred at the event point; solid blue line, projected slope over guideline period 2.

Over the full study in period, in the ED group, adjusted use decreased from 56.3% (95% CI, 51.1% to 61.5%) to 26.0% (95% CI, 20.8% to 31.2%) for bronchodilators, 15.7% (95% CI, 13.0% to 18.2%) to 6.1% (95% CI, 3.4% to 8.7%) for steroids, and 4.2% (95% CI 3.3% to 5.0%) to 2.7% (95% CI, 1.8% to 3.6%) for antibiotics. In the inpatient group, adjusted use decreased from 73.0% (95% CI, 68.0% to 78.0%) to 50.1% (95% CI, 45.1% to 55.1%) for bronchodilators, 38.6% (95% CI, 35.3% to 42.0%) to 17.9% (95% CI, 14.6% to 21.2%) for steroids, and 49.1% (95% CI, 46.9% to 51.2%) to 23.8% (95% CI, 21.6% to 25.9%) for antibiotics. Trends in treatment measures over time are shown in Figure 2.

### Balancing Measures

Over the full study period, LOS decreased from 2.0 days (95% CI, 1.9 days-2.1 days) to 1.7 days (95% CI, 1.7 days-1.8 days), with a negative slope over both guideline periods. An increase of 0.03 days (95% CI 0.02 days-0.08 days) was found at the event point; there was no change in slope between periods (Table 2). Hospital admission rate decreased over the full study period, from 18.0% (95% CI, 13.8%-22.2%) to 17.8% (95% CI, 13.6%-22.1%). There was a positive change in slope of 0.02% (95% CI, 0.01%-0.03%) over guideline period 2; no change in hospital admission rate was found at the event point (Table 2).

## Discussion

This cohort study found that in the 13 years since publication of the 2006 AAP bronchiolitis guideline, there has been a consistently declining trajectory in most overuse measures in a sample of US children’s hospitals. After the 2014 guideline publication, there were further decreases in the ED and inpatient groups in use of CR, viral testing, bronchodilators, and steroids. There was also a significant trajectory change in bronchodilator use after the 2014 update, with rates of decrease more than 2-fold those observed before the update in both groups. These findings occurred in the context of small changes in LOS and hospital admission rates over the study period that are unlikely to represent a clinically relevant change in outcomes.

Given that the largest changes over time were found for the bronchodilator measure, these changes warrant further discussion. The 2014 guideline update^1^ states that bronchodilators should not be administered to patients with bronchiolitis, replacing statements in the 2006 guideline^6^ that allowed for a monitored bronchodilator trial among these patients. The albuterol recommendation was the most substantive change between the 2 publications; thus, it may not be surprising that bronchodilator use is the measure for which the greatest change was observed following the 2014 update. While ITS analysis separates change into an incremental component and a slope component, we recognize that our large data set with a longer time period may create the appearance of a sudden change, which in reality was more gradual. However, the significant change in the trajectory of bronchodilator use after the 2014 guideline update further supports our choice of event point.

Significant knowledge gaps remain surrounding how practice guidelines translate into clinical improvements. There is evidence to suggest that characteristics of guideline recommendations may be associated with the success of guideline uptake.^14^ The 2006^1^ and 2014^6^ bronchiolitis guidelines focused on reducing tests and treatments for which there is no supportive evidence. This process, also referred to as deimplementation, is associated with particular challenges for clinicians due to structural and psychological barriers associated with discontinuation of existing practices.^15,16^ Whereas providers may adopt practices based on relatively weak early evidence for benefit, there is often resistance to reversal of practices until strong evidence for inefficacy is found.^17^ Our study results support the idea that a nationally promulgated clinical practice guideline may help overcome some barriers to deimplementation.

One mechanism through which clinical practice guidelines may facilitate deimplementation at the patient level is by supporting active quality improvement (QI) interventions. A 2014 systematic review^18^ of 14 published QI initiatives in inpatient bronchiolitis care found that these interventions were associated with reductions in bronchodilator, steroid, antibiotic, and CR use. The QI efforts included in this review or published after the review have been associated with reduced use of nonrecommended services by implementing local clinical pathways citing AAP recommendations^19,20,21,22,23,24,25,26,27,28,29^; international studies using other national guidelines as the basis for local improvements have been associated with similar improvements.^30,31,32,33^ Such QI efforts constitute 1 factor potentially associated with the decline in nonrecommended services found across our study period.

While we suggest that our study provides evidence supporting the association of clinical guidelines with improved bronchiolitis care, there are many additional factors that were likely associated with changes in care patterns over our study period. Awareness of health care overuse as a widespread problem has increased over this time period, and other published recommendations focused on reducing low-value practices have followed. One such example is the Choosing Wisely campaign, an initiative founded by the American Board of Internal Medicine in 2012 to promote evidence-based, necessary care.^34^ In 2013, several recommendations for bronchiolitis were added to the Choosing Wisely initiative, including those recommending against the use of steroids, bronchodilators, CRs, and antibiotics.^35,36^ Discriminating the outcomes associated with such important initiatives from those associated with clinical guidelines is challenging. The AAP guidelines are cited as support for these Choosing Wisely measures, and it seems likely that guidance from varying sources are associated with synergistic outcomes.^35,36^

Our study found important improvements in bronchiolitis care in the ED and inpatient settings. However, usage rates observed at the end of our study period also suggest a need for continued deimplementation efforts. Higher rates of use observed in the inpatient group compared with the ED group may be associated with increased intervention for patients with more acute presentations, and this may or may not be warranted. Increased focus on developing and adjusting realistic improvement targets, such as achievable benchmarks of care, may play an important role in future deimplementation efforts.^37^ Appropriate usage rates for the measures evaluated in this study are not always zero; notably, however, rates at the end of our study period remain well above achievable benchmarks of care previously established using PHIS data, particularly in the inpatient group.^3,38,39^

### Limitations

This study has several limitations. We used administrative data to determine our study population and use patterns. Administrative data do not provide detailed clinical information for each encounter, and therefore we cannot determine which clinical scenarios or levels of acuity may be associated with use of measured services. Our population included only patients cared for in US children’s hospitals, which deliver a minority of bronchiolitis care.^40^ Therefore, our findings may not be generalizable to other care settings. For the inpatient group, we were unable to differentiate care provided in the ED prior to hospitalization from care delivered in the inpatient setting. There are 2 issues associated with seasonality that relate to our analysis. First, bronchiolitis severity fluctuates from year to year, and we did not adjust for severity of illness at the patient level; however, we included a long time series in which year to year variation in disease severity should balance out over time. Second, we did not adjust for the known variation in use of these tests and treatments associated with variability in patient volume over the course of any given year^41,42^; however, we included monthly use rates in our regression model and a similar number and distribution of months in the 2 guideline periods to minimize the association of seasonality factors with our results. We cannot establish a direct causal relationship between guideline publication and the trends observed in this study. The use of segmented regression analysis supported a temporal association; however, we did not evaluate other secular trends that may have been associated with changes in bronchiolitis care over this time period. As noted, dichotomizing the long inclusion period may overestimate the magnitude of the level change associated with the event point.

## Conclusions

This cohort study found that use of nonevidence-based tests and therapies for bronchiolitis has decreased since 2006 in a sample of encounters at US children’s hospitals. Publication of the 2014 AAP bronchiolitis guideline update was temporally associated with further decreases in use of some nonrecommended services, with the greatest decrease observed for bronchodilator use.
